# Topologically-optimized on-chip metamaterials for ultra-short-range light focusing and mode-size conversion

**DOI:** 10.1515/nanoph-2023-0036

**Published:** 2023-02-27

**Authors:** Wei Ma, Maojing Hou, Ruiqi Luo, Bo Xiong, Nan Liu, Guandong Liu, Tao Chu

**Affiliations:** State Key Laboratory of Modern Optical Instrumentation, College of Information Science and Electronic Engineering, Zhejiang University, Hangzhou 310027, China; Zhejiang Lab, Intelligent Network Research Institute, Hangzhou 311100, China

**Keywords:** integrated photonics, inverse design, meta-lens, metamaterial

## Abstract

The concept of metamaterials offers a flexible pathway to manipulate the macroscopic behavior of light by delicately designed microscopic subwavelength structures, which has been recently introduced to integrated photonics to create devices with ultra-compact footprint, excellent performance or versatile functionalities. However, the conventional design approach of metamaterials, including two separated steps of subwavelength structure design and the assembly of unit cells, often encounters challenges when facing extreme design targets. In this work, we propose a hierarchical inverse design approach by cascading a conventional unit-cell-based design with a holistic topology optimization. As a proof-of-concept, we demonstrate ultra-short-range light focusing and mode-size conversion enabled by on-chip meta-lenses. The shortening of tapering region pushes higher numerical aperture of on-chip lenses, leading to the violation of locally periodic approximation used in meta-lens design and thus poor device performance, which fortunately, can be well compensated by the follow-up holistic optimization step. We experimentally realize mode-size squeezing by almost 20 times in a tapering region as short as 8 μm and 5 μm with low insertion loss and broadband performance. The proposed design scheme provides practical guidelines to design metamaterials as flexible on-chip wavefront control and light routing devices for various applications in fiber communication, sensing and optical computing.

## 1 Introduction

Similar as electronic integrated circuit, there has been a long pursuit in integrated photonics research to shrink the size of devices and improve the integration density of systems. Silicon is a promising integrated optic platform due to its good properties at optical communication band, and more importantly, its compatibility with complementary metal-oxide semiconductor (CMOS) fabrication processes [1]. One bottleneck in increasing the integration density of silicon photonic circuits is the size of the ever-increasing light routing structures as the number of integrated optical devices scale up. For instance, in components like grating couplers, multimode interference couplers, arrayed waveguide gratings and waveguide crossings, mode-size conversion is required to connect two waveguides with different widths while keeping the fundamental mode when light propagates through. Typically, such working condition is usually guaranteed by the so-called adiabatic taper structure where the cross-section of the taper varies very slowly along the propagation direction to prevent the excitation of higher-order modes. With a conventional linear boundary, a taper often has a total length over 300 μm to transform adiabatically from a 10 μm grating coupling region to a conventional 500 nm single-mode waveguide [2]. Even with specially designed line shape of exponential [3], parabolic [4], or Gaussian expansion [5], the length of an adiabatic taper can hardly be reduced below 100 μm. Such long taper structures pose significant challenges in densely integrated photonic devices and systems. The biggest problem of a conventional mode-size converter is that an empirical line shape is artificially assigned in advance to define only the boundary of the tapering region, which requires a follow-up parameter-sweep-based optimization that is not flexible enough to scale down the design further [6].

Metamaterials and metasurfaces, which are composed of arrays of subwavelength structures, offer unprecedented degree of freedom in photonic design to manipulate the amplitude, phase, frequency and polarization of light [7–9]. More recently, the concept of metamaterials has been introduced to integrated photonics as meta-waveguide devices to manipulate both on-chip and out-of-chip light field [10–14], spawning many compact and flexible design of silicon photonic devices such as wavelength demultiplexer [15], mode converter [16, 17], edge coupler [18] and so on. In the context of mode size conversion, a common metamaterial-based approach is to use the constituent subwavelength structures as effective media, forming an on-chip gradient index focusing lens that converts large mode profile to fit single mode waveguide [19–22]. However, these designs still rely on the propagation of light to accumulate phase change, which hinders the scale-down of the tapering region. Another approach is to directly adapt the concept of two-dimensional planar meta-lens to one-dimensional version on an integrated photonic platform [23]. By employing a series of nano-slots that introduce abrupt phase changes, flexible on-chip wavefront shaping can be realized with functions such as lensing and Fourier transformation [24]. With such meta-lens-based design principle, a mode-size converter with 13.7 μm-long tapering region was demonstrated. Nevertheless, limited by the locally periodic approximation in meta-lens design, high numerical aperture that is required by an ultra-short tapering region can hardly be achieved in the naïve meta-lens design approach [25].

To further unleash the potential of metamaterials and meta-lenses, inverse design methods become popular to enable on-demand design and push the performance of conventionally designed devices to extremes [26–28]. Meta-lenses with high efficiency, multi-functionality and large numerical aperture were successfully demonstrated using inverse design algorithms [29–33]. For integrated photonics, inverse design methods are exploited to produce devices with ultracompact footprint and versatile functionalities, such as wavelength demultiplexers [34, 35], polarization splitters [36], mode converters [37], microresonators [38], beam steerers [39], mode sorters [40] and so on. In most cases, the inverse design of these ultra-compact metamaterial-based integrated photonic devices starts from a randomly initialized device with continuous refractive index distribution, which gradually converges to binary structure with unconventional topology as the optimization proceeds. This routine is not efficient enough and can be easily trapped in undesired local optima.

In this work, we leverage the concept of meta-lens and inverse design method to demonstrate ultra-short-range on-chip light focusing and mode-size conversion with tapering region well below 10 μm. The design follows a hierarchical approach to first construct an on-chip metamaterial lens that focuses light directly from wide waveguide into the narrow end. Then level-set-method-based topology optimizations are applied to the initial metamaterial structure to compensate for the negative effect of the violation of locally periodic approximation in the design of high numerical aperture meta-lens, further improving the device performance. We make a comparative study and experimentally demonstrate efficient, broadband mode-size converters with a length of 5 μm and 8 μm. The proposed hierarchical scheme is not only limited to mode-size converter design, but also offers a flexible tool for other integrated photonic structures that routes guided light with ultra-compact footprint.

## 2 Overall design pipeline

**Figure 1:**
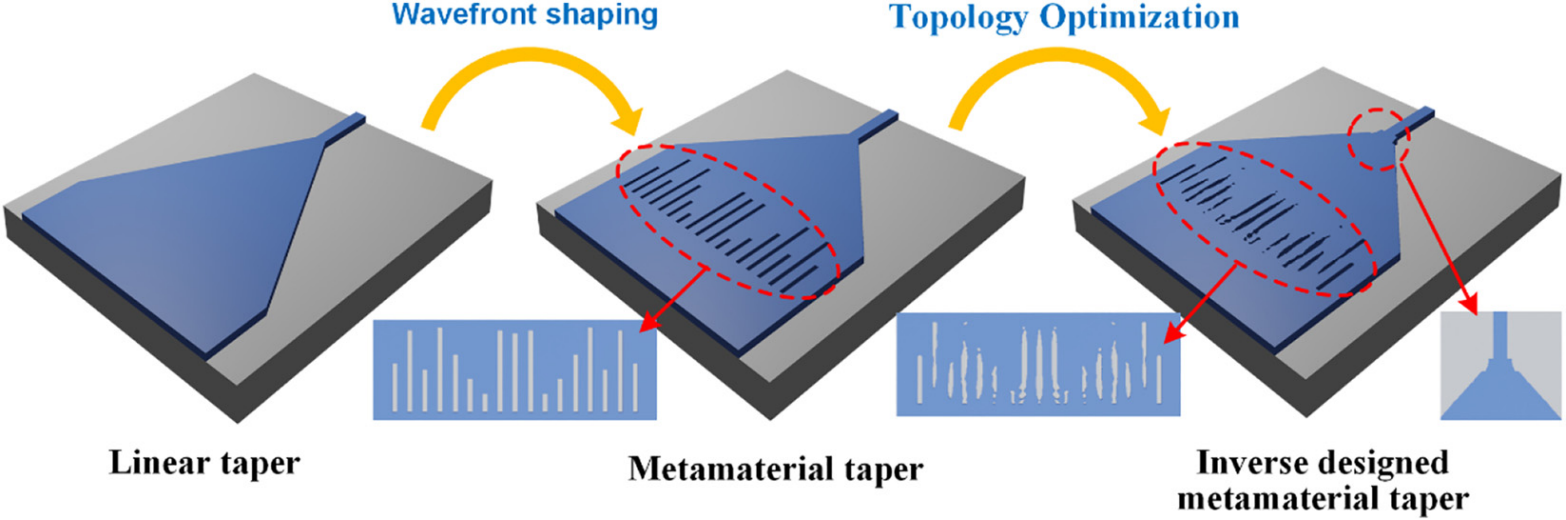
Hierarchical design scheme of ultra-short metamaterial mode-size converter.

The design of ultra-short metamaterial mode-size converters follows a three-step hierarchical scheme as shown in Figure 1. Initially, a linear taper is directly applied to connect the wide waveguide and narrow waveguide. In such case, adiabatic transition requires that the spreading of the waveguide sidewalls is slower than the diffraction spreading of the first-order mode to prevent possible mode conversion to higher-order modes or radiation modes [41]. As the length of a linear taper decreases, the adiabatic transition condition is gradually destroyed, so that the tapering region is unable to confine light properly, leading to low coupling efficiency. To overcome the limit of adiabatic taper length, artificial photonic structures, namely metamaterials, are introduced as wavefront shaping region, which are composed of an array of rectangular slots. The on-chip metamaterials functions as a focusing lens that funnels light from the wide waveguide to the narrow end. When the taper length continues to decrease, the conventionally designed metamaterials with necessary approximations and regular shapes are unable to function efficiently. To further boost the coupling efficiency, topology-optimization methods are employed to modify the entire device, including both metamaterial region and the taper boundaries. With this secondary optimization process, the rectangular metamaterial slots evolve to irregular geometries with slightly modified taper boundaries. This new topology overcomes the limitation of the locally periodic approximation, which is usually unable to fulfill in the design of meta-lens with very high numerical aperture.

## 3 On-chip meta-lens design

**Figure 2:**
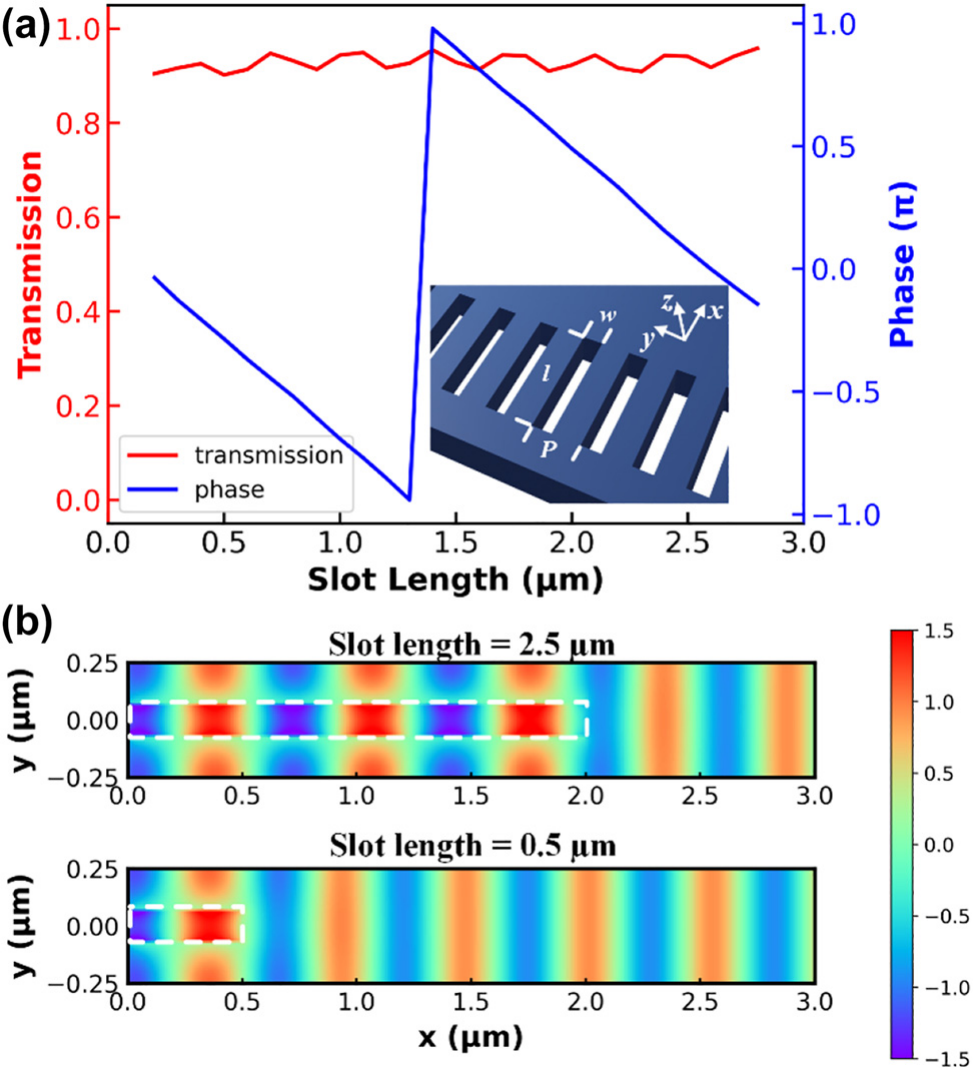
Design of the unit cell of the on-chip metamaterial. (a) Transmission and phase difference of metamaterial unit cell as a function of slot length. (b) Distribution of *y* component of electric field (*E*_*y*_) at the center wavelength of 1550 nm with two different slot width of 0.5 μm and 2.5 μm.

Given the desired device length, the first step is the design of an on-chip meta-lens as a wavefront shaping structure with focal length that matches the taper length. We employ a silicon on insulator (SOI) platform with 220 nm-thick silicon layer, 3 μm-buried oxide and SiO_2_ cladding layer. We use the rectangular void slots as phase shifting unit cell [24]. The width of the slot is fixed at 140 nm with a period of 500 nm along *y* axis, and the length is varied to introduce required phase shift at the working wavelength of 1550 nm. As shown in Figure 2(a), when the slot lengths increase from 0.2 μm to 2.8 μm, the transmissions remain above 0.9 while the phase change, observed at a monitor point 3 μm away from the slot far end, covers the entire 2*π* range. It should bed noted that by increasing the width of the nano slot, the 2*π* phase tuning can be achieved at shorter slot width, but will cause higher scattering and thus lower transmission efficiency. Therefore, the width of 140 nm is chosen as a compromise between phase tuning efficiency and transmission efficiency.

The simulation of the unit cell structure is conducted with periodic boundary condition and transverse electric (TE) mode excitation. To further unveil the mechanism of such phase shift, we pick two slot lengths, namely 0.5 μm and 2.5 μm, and calculate the *y* component of electric field distribution at the cutting plane in the middle of the unit cell, as depicted in Figure 2(b). The deep subwavelength slot width of 140 nm introduce little scattering of the incident light but behaves like an effective media, yielding high transmission [10]. The effective index in the metamaterial region is controlled by the slot length, where longer slot leads to lower effective index and thus a phase retardation accordingly.

To introduce wavefront shaping metamaterial structures to shorten the taper region, we need to construct an on-chip meta-lens with predefined focal point where the narrow end of the taper is located. Therefore, a hyperbolic phase profile is enforced along *y* direction, as shown in Eq. (1). We use 17 metamaterial slots covering a total width of 9.5 μm of the wide waveguide, whose lengths are determined by the phase requirement and the phase-length relation in Figure 2(a).

(1) $$\Delta \varphi \left(x\right)=\frac{2\pi }{\lambda }\left(\sqrt{{\left(x-\Delta x\right)}^{2}+f^{2}}-f\right)$$

**Figure 3:**
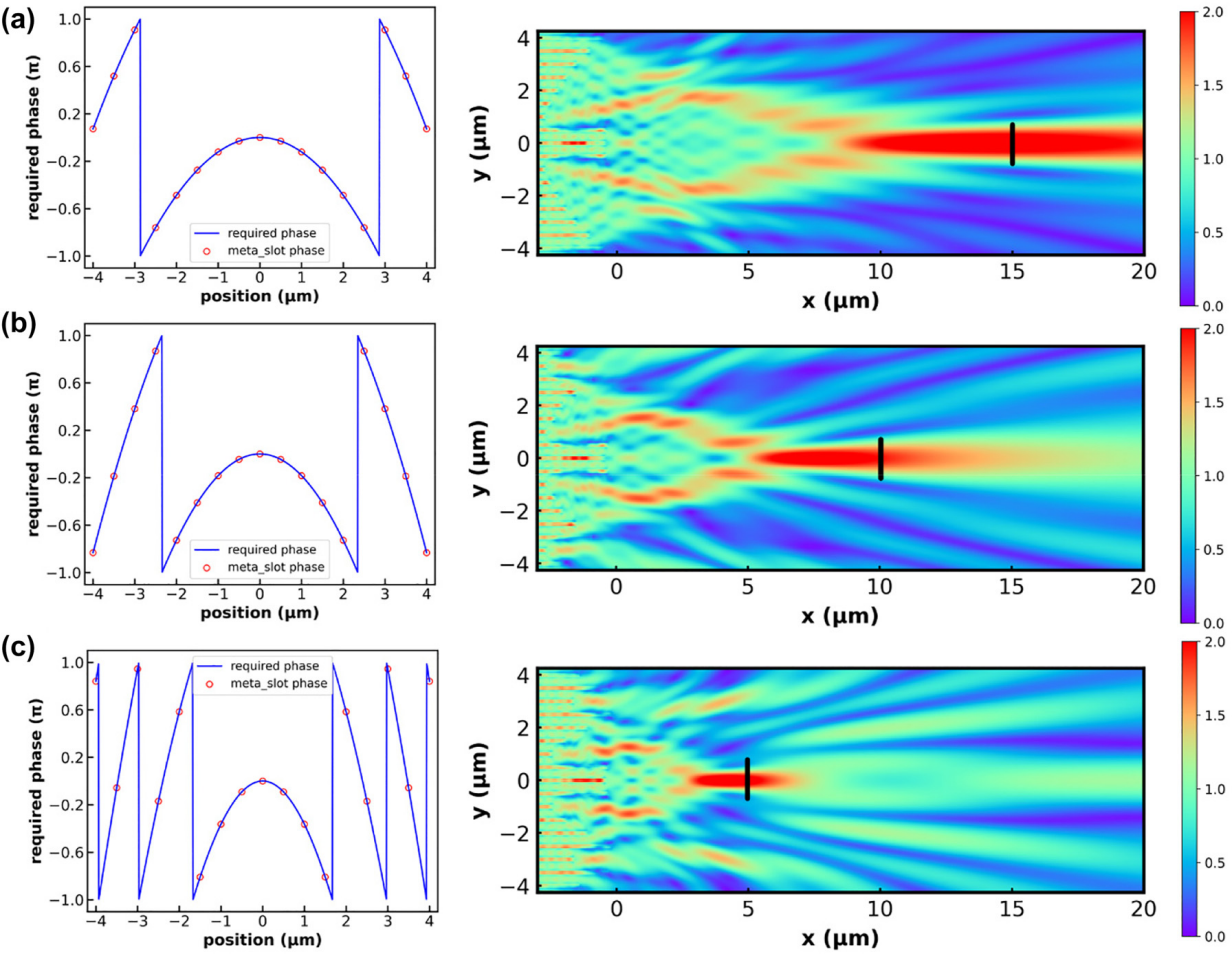
Phase distribution and electric field intensity of the on-chip meta-lenses with focal length of 15 μm (a), 10 μm (b) and 5 μm (c) respectively. The blue line is the ideal phase distribution along *y* direction, where the red hollow circles indicate the chosen phase at the exact slot position. The black lines at the focal point in the field distribution plot indicate the cutting plane to calculate the focusing efficiency. The focus efficiency is 0.317, 0.402 and 0.577 for the focal length of 5 μm, 10 μm and 15 μm, respectively.

To demonstrate the limitation of such traditional meta-lens design for on-chip applications, we pick 3 different focal lengths, namely 5 μm, 10 μm and 15 μm. The required phases are plotted in Figure 3 as blue curves with red hollow circles denoting the exact phase at each discrete location of metamaterial slot. It is clear that when the focal length decreases, larger variation of the phase difference is required to form a lens with higher numerical aperture. We also calculate the electric field distribution to straightforwardly illustrate the focusing effect. We notice that a small deviation of the focal point appears as the focal length decreases. As indicated by the black line, we set a 1.5 μm wide monitor at the designed focal point to obtain the focusing efficiency of the on-chip lens, which turn out to be 0.577, 0.402 and 0.317 for the lens with focal length of 15 μm, 10 μm and 5 μm, respectively. The primary reason for such plunge in focusing efficiency and the deviation of focal point is the locally periodic approximation applied in meta-lens design. In the previous design procedure, the phase change of each metamaterial slot is obtained under periodic boundary condition, which means that in the construction of a meta-lens, the size of adjacent slots and thus the transmitted phase must vary very slowly to guarantee the accuracy of design. Such approximation is severely violated as the numerical aperture of the meta-lens continues to increase.

## 4 Topology optimization of the device

In order to compensate for the negative effect originated from the locally periodic approximation, we further applied topology optimization on the initial meta-lens-based mode-size converter design. Since the meta-lens design provides a starting point with moderate performance, the optimization is directly applied on the discrete structure with level set representation. The design region is a two-dimensional structure composed of only two materials, silicon and silicon dioxide. To introduce level set representation, we construct a continuous function $\phi \left(x,y\right)$ that maps *x* and *y* coordinates into a continuous value, and makes the boundaries between the materials lie on the zero level set $\phi \left(x,y\right)=0$ . Then the permittivity distribution is given by

(2) $$\varepsilon \left(x,y\right)=\left\{\begin{array}{c} \varepsilon_{\text{Si}}\;for\;\phi \left(x,y\right)\leq 0\;\\ \varepsilon_{\text{Si}02}\;for\;\phi \left(x,y\right)>0\; \end{array}\right.$$

The biggest advantage of the implicit level set representation is that it can conveniently deal with complex morphological changes such as merging or splitting of holes. To optimize our device, we first choose an appropriate target function *f* $\left(\varepsilon \right)$ , and evolve the level set function $\phi \left(x,y\right)$ to maximize the target. This is realized by first applying adjoint sensitivity analysis to calculate the gradient of the target function with respect to the permittivity distribution, then setting the velocity term in the level set equation to be proportional to the gradients. A curvature radius limitation of 100 nm is employed during optimization, so any sharp corners with curvature radius smaller than 100 nm will gradually be smoothed during optimization [42]. We also periodically employ erosion and dilation operation, as a routine in image processing, to remove possible narrow bridges or gaps that my occur during level-set based optimization. Detailed information about the inverse design process are described in the method section.

**Figure 4:**
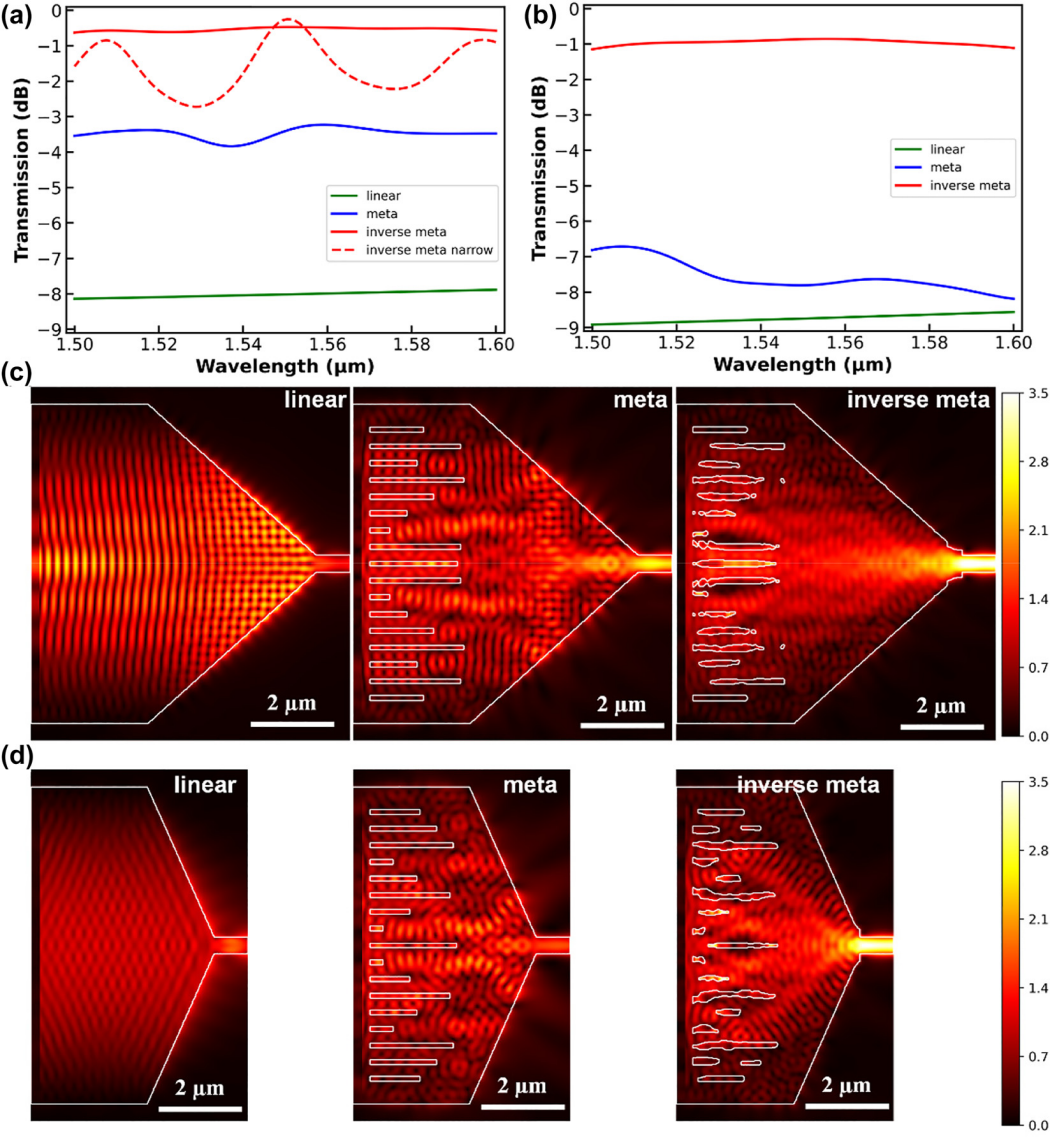
Simulated insertion loss of the mode-size converters with length of 8 μm (a) and 5 μm (b), respectively. The green lines show the simple linear taper without metamaterial structures. The red lines and blue lines plot the metamaterial mode-size converters with only manual design and after inverse design is applied. The red dashed line in (a) is the optimization results without using a broadband target function. The square of the electric field intensity ( ${\left|E\right|}^{2}$ ) distribution for 8 μm device (c) and 5 μm device (d).

Since the initial meta-lens design resort to the effective index variation from the void slots, which is independent of the working wavelength, the metamaterial mode-size converters have a broad working band, as shown by the blue cures in Figure 4(a) and (b). During inverse design, we choose the target function to be the mean transmission from 1500 nm to 1600 nm instead of the transmission only at central wavelength of 1550 nm, namely,

(3) $$f\left(\varepsilon \right)=\frac{1}{N}\overset{N}{\underset{\lambda \in \left(1500,\;1600\right)}{\sum }}f\left(\varepsilon ,\lambda \right)$$

In our implementation, we uniformly pick *N* = 20 wavelength points between 1500 nm and 1600 nm and optimize the target function in Eq. (3). Figure 4(a) demonstrates comparative results of a mode-size converter with length of 8 μm. Since the phase changes in Figure 2(a) are obtained by the void slots in a 3 μm long unit cell, the mode-size converting structure will have an intrinsic length of 3 μm. The initial design of an 8 μm long mode-size converter is obtained by creating a meta-lens with focal length of 5 μm, and directly connecting the 500 nm single mode waveguide and the input 9.5 μm wide waveguide by linear boundaries. A broadband transmission of about −4dB to −3dB is observed (blue curve). In contrast, if no metamaterial is introduced, a simple 8 μm long linear taper has a very low transmission of −8dB (green curve).

After inverse design with broadband target function, the overall transmission is raised to the level around −0.5 dB (solid red curve). If the optimization is conducted only at the center frequency of 1550 nm, a very high peak of −0.2 dB is observed at the center frequency, but the transmission spectrum oscillates and drops to near −3dB at some wavelength. Therefore, the broadband inverse design increases the transmission by over 3 dB over the entire C band compared with the manually designed metamaterial mode-size converter. The different transmission spectra are also verified by the field distribution results shown in Figure 4(c), where a great portion of energy is reflected from or transmitted out of the boundaries in the simple linear taper, causing many ripple patterns in the field distribution. These energy losses are alleviated by introducing the metamaterial structures to focus incident light into smaller size and then funnel to the narrow end. The inverse designed structure further improves the transmission by compensating for the non-ideality in meta-lenses, resulting in the highest field intensity in the output waveguide.

To further verify the potency of the proposed design scheme, we reduce the device length down to 5 μm enabled by a meta-lens with a focal length of only 2 μm. In this case, the design principle of meta-lens with locally periodic approximation is completely violated. As plotted in Figure 4(b), both the conventional linear taper and manually designed metamaterial mode-size converter exhibit very low transmission. For a predefined focal length of 2 μm, the assigned metamaterial slots fail to form an effective focusing lens but act mere as scatterers that contribute little to the coupling efficiency. Similarly, when the broadband inverse design method is applied, the transmission rises to about −1dB over the entire C band, manifesting the capability of the proposed hierarchical design scheme.

In the topologically optimized device, some fine features, such as small holes, are observed. To investigate whether these fine features are critical for the device performance, we employ sensitivity analysis to calculate the gradient of the target function with respect to the permittivity distribution of the design region. The normalized gradient along with the electric field distribution is shown in Figure S1 (See supplementary material for more information). Larger gradient value means the permittivity at that point will produce higher influence on the overall design target, and the most sensitive region in the device is located where the electric field is most intensity. Generally, most fine features in the optimized device are not very critical for the overall performance, which exhibit strong design robustness and fabrication tolerance.

## 5 Experiments and discussions

**Figure 5:**
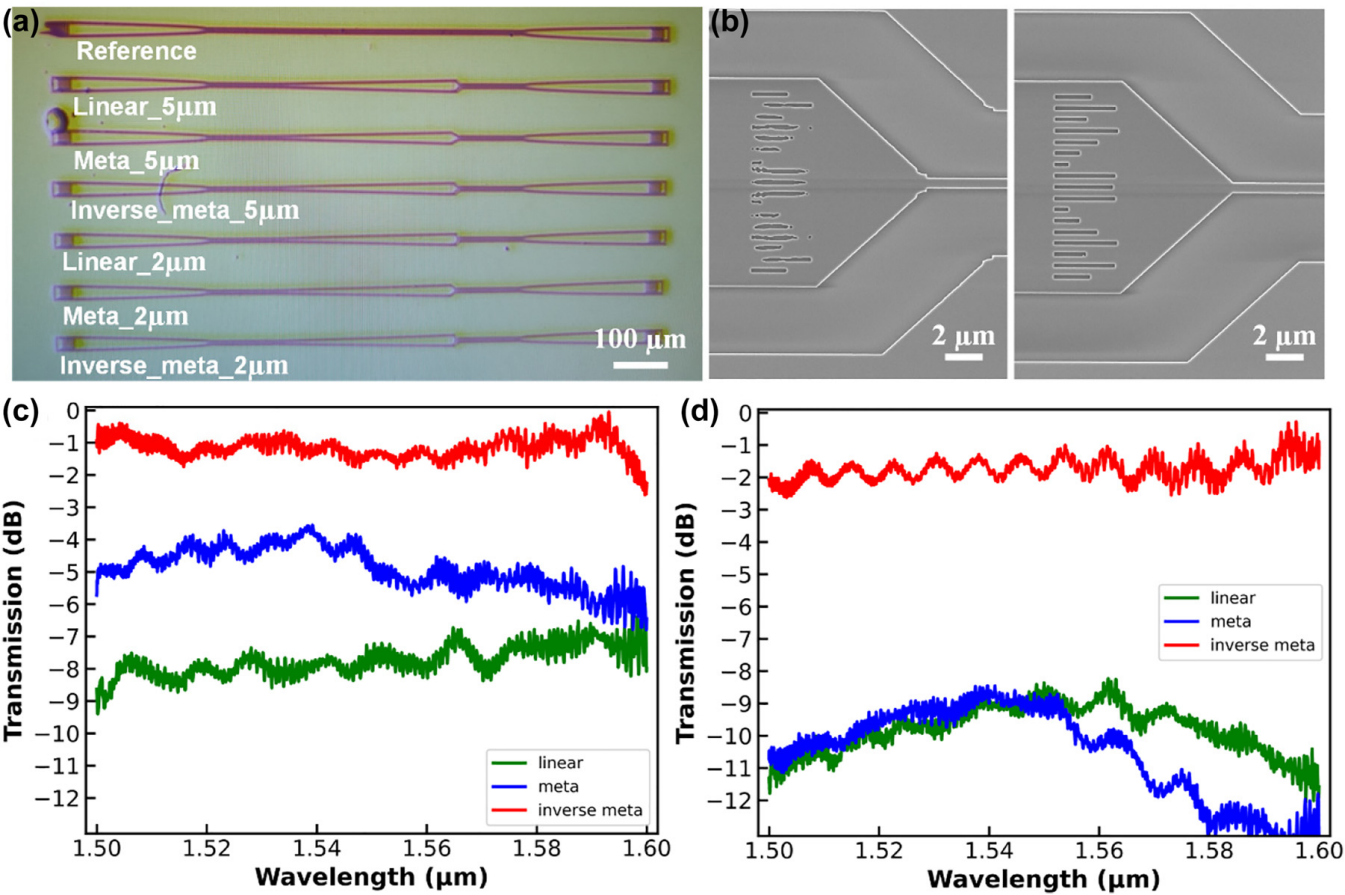
Experimental measurement of the fabricated devices. (a) Optical microscopic picture of the fabricated devices. (b) Scanning electron microscope (SEM) image of the fabricated 8 μm mode-size converter with and without inverse design. The experimentally measured transmission over the entire C band for devices with length of 8 μm (c) and 5 μm (d), respectively.

To experimentally verify the designed device, we fabricated a series of mode-size converters for a comparative study. Detailed fabrication processes are discussed in the method section. We fabricated simple linear tapers, manually designed metamaterial mode-size converters and inverse-designed metamaterial mode-size converters with the lengths of both 8 μm and 5 μm as simulated in Figure 4. Figure 5(a) shows the optical microscope picture of the fabricated devices. We use two grating couplers to couple incident light into and out of the chip, each of which is connected by a 300 μm linear adiabatic taper to transform the guided light into a 500 nm single mode waveguide with fundamental TE mode. The mode-size converter lies in between the two grating couples, with the narrow end connected to the grating directly and the wide end connected to the grating via another 300 μm adiabatic taper. The 300 μm adiabatic taper is long enough to transform the light with fundamental TE mode from the 9.5 μm waveguide to the 500 nm waveguide, which guarantees single mode wave propagation with low loss. We also set a reference group with only two grating couplers connected by a section of 500 nm waveguide with the same total length as the testing device to cancel out the coupling loss. Figure 5(a) demonstrates the SEM images of the 8 μm devices with and without inverse design, showing good fabrication accuracy.

The measured transmission spectra are plotted in Figure 5(c) and (d) for 8 μm and 5 μm device respectively. Obviously, inverse designed metamaterial mode-size converters outperform the conventional linear tapers and manually designed metamaterial devices by a large margin. After normalization by the reference grating, the 8 μm inverse designed metamaterial mode-size converter has a wide operating band from 1500 nm to 1600 nm with an average insertion loss of around −1dB, while the 5 μm device also delivers wideband performance with around −2dB insertion loss. The experiment agrees well with the previous simulation, verifying the effectiveness of the proposed hierarchical inverse design scheme.

## 6 Conclusions

In summary, we propose a hierarchical inverse design scheme for on-chip metamaterials, and experimentally demonstrate ultra-short-range light focusing and broadband mode-size converters with total lengths of only 5 μm and 8 μm. By first constructing a meta-lens in the input region of the taper with the focal point at the narrow waveguide end, a manually designed metamaterial structure is created with coupling efficiency much higher than the simple linear taper. In order to compensate for the violation of locally periodic approximation used in conventional meta-lens design, we apply a holistic topology optimization right on the manual design in the second step, further pushing the device performance by a large margin. Compared with the popular end-to-end inverse design from randomly initialized permittivity distribution, the proposed hierarchical design scheme utilizes the conventional unit-cell-based metamaterial design as a bridge, which facilitates large area optimization in a more efficient way with less risk of undesired local optima. The hierarchical design scheme is not only limited to mode-size converter design, but is flexible enough to extend to other metamaterial structures for on-chip wavefront shaping and ultra-compact light routing, including the manipulation of wavelength, polarization and mode. These metamaterial-based devices offer ultra-compact and versatile solution for various applications such as optical fiber communication, portable optical sensing platform, optical computing chips and integrated optical information processing systems.

## 7 Methods

### 7.1 Inverse design details

To conduct the broadband inverse design on the meta-lens structure, we use level set and adjoint optimization codes written in python, which is coupled to the commercial Lumerical FDTD simulation tool. Unlike other popular implementations of adjoint method using FDFD backbone [43], time domain simulation can produce results over a broadband range other than a single wavelength, and is more scalable to larger simulation domain as the case of our work. The source and the field monitors are set to cover wavelength raging from 1500 nm to 1600 nm, from which we simultaneously obtain the gradient information at the uniformly spaced 20 wavelength points through two simulations, namely a forward simulation and an adjoint simulation. During simulation, symmetric condition is enforced with respect to *y* axis to expedite the optimization process. After the gradients are obtained, we evolve the level set function according to the below equation:

$$\varphi_{t}\left(x,y\right)+\nabla f\left(\varepsilon \right)\cdot \left|\nabla \varphi_{t}\left(x,y\right)\right|=0$$

where $f\left(\varepsilon \right)$ is the target function with respect to the permittivity distribution. The above level set equation is discretized with a time step following Courant–Friedrechs–Lewy condition [44] with spatial grid size Δ*x* and a scale factor of *α* = 0.9, that is:

$$\Delta t=\frac{\alpha \cdot \Delta x}{{\left|\nabla \varphi_{t}\left(x,y\right)\right|}_{\text{max}}}$$

The level set function *φ* is periodically re-initialized to the signed distance function every 5 iteration to stabilize the optimization process. The overall optimization takes 60 iterations before reaching steady state with optimal design.

### 7.2 Device fabrication

The devices were fabricated on a silicon-on-insulator substrate with 220 nm silicon device layer and 3 μm underlying buried oxide. To form the entire testing devices, we first fabricate the grating coupler with shallow etching of 70 nm. The grating patterns were written with electron-beam lithography (EBL) system and ZEP520A as the resist. After development of the pattern, inductively coupled plasma (ICP) etching was used to etch the silicon layer by 70 nm. Then we repeat the EBL and ICP process to define the metamaterial structure with a full etching depth of 220 nm, which is aligned with the previously defined grating structure. Finally, another layer of SiO_2_ was grown as cladding layer using plasma enhanced chemical vapor deposition (PECVD) technique after the resist was removed in acetone.

## Supplementary Material

Supplementary Material Details
