# Supplementary material for: Topologically-optimized on-chip metamaterials for ultra-short-range light focusing and mode-size conversion
Source: Nanophotonics. 2023 Feb 27;12(6):1189–97. doi: 10.1515/nanoph-2023-0036 (PMC11501792; doi:10.1515/nanoph-2023-0036)
Supplement: Supplementary file 1 — Supplementary Material Details [file j_nanoph-2023-0036_suppl_001.pdf]

Supplementary Materials for
Topologically-optimized on-chip metamaterials for ultra-short-range light focusing and mode-size conversion

Wei Ma[1,2,*], Maojing Hou[2], Ruiqi Luo[2], Bo Xiong[1], Nan Liu[2], Guandong Liu[2] and Tao Chu[1,2]

[1] State Key Laboratory of Modern Optical Instrumentation, College of Information Science and Electronic Engineering, Zhejiang University, Hangzhou 310027, China.
[2] Intelligent Network Research Institute, Zhejiang Lab, Hangzhou 311100, China.
*Corresponding author: ma_wei@zju.edu.cn

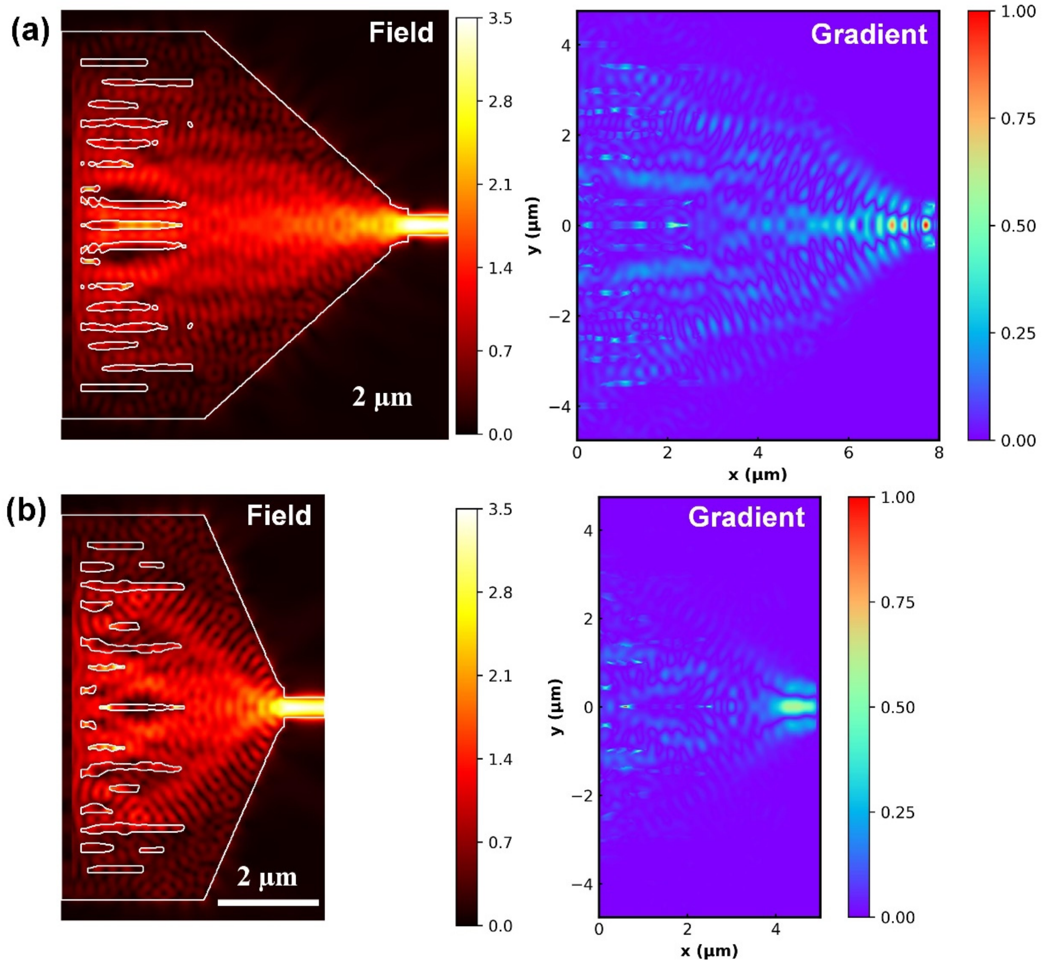

Figure S1: Field distribution and normalized gradient distribution of the inverse-designed metamaterial mode-size converter. (a) 8µm device, (b) 5µm device.
